# Incidence and trends in workplace violence within emergency departments in the United Kingdom 2017–2022: an observational time series analysis

**DOI:** 10.3389/fpubh.2023.1211471

**Published:** 2023-06-28

**Authors:** Neil Donald, Tim Lindsay

**Affiliations:** ^1^Department of Surgery, Dartford and Gravesham NHS Trust, Dartford, United Kingdom; ^2^Department of Trauma and Orthopaedics, London North West University Hospitals NHS Trust, London, United Kingdom; ^3^Imperial College London, London, United Kingdom

**Keywords:** staff safety, emergency medicine, healthcare workers, occupational health, COVID-19, healthcare policy, workplace violence

## Abstract

**Background:**

Workplace violence (WPV) is a notable issue facing healthcare services and workers globally. WPV impacts upon the well-being of staff and can put healthcare provision at risk with detrimental effects on patient care. This study aims to investigate and quantify, at national and regional levels, the incidence and trends of WPV within emergency departments (EDs).

**Methods:**

We requested data relating to WPV from all 152 trusts with an ED in the United Kingdom from January 2017–March 2022. We applied interrupted time series and trend analysis to check for significant differences in WPV across the COVID-19 pandemic.

**Results:**

We conducted time series analysis on 58 million attendances and detected statistically significant increases in WPV in March 2020–5.06/100,000 attendances (95% CI 1.59/100,000–8.53/100,000 *p* < 0.01) and May 2020–20.63/100,000 attendances (95% CI 9.39–31.87 *p* < 0.01). Rises in incidents of 0.37/100,000 attendances per month (95% CI 0.21–0.53 *p* < 0.0001) were found January 2017–March 2020. We analyzed 96 million attendances for yearly trends, which revealed statistically significant increasing trends of WPV in London and North-West England (*p* < 0.05), and physical WPV in the North West England (*p* < 0.05).

**Conclusion:**

There have been dramatic increases in incidents of WPV in United Kingdom EDs over the last 5 years with concerning rises during the COVID-19 period. Our findings highlight the potential to further demoralize a workforce already under significant strain, resulting in increased absences for physical or mental health and an exodus of staff. Therefore, trusts should ensure there are robust systems in place to protect and safeguard staff.

## Introduction

Workplace violence (WPV) against healthcare workers (HCWs) is a notable and widespread healthcare issue with a high global prevalence (1). WPV is an umbrella term that is defined by the World Health Organization (WHO) as “incidents involving work-related abuse, threats or assaults among HCWs including physical, sexual, verbal, psychological abuse and workplace harassment.” (1) The World Medical Association (WMA) has condemned the increase in cases of WPV perpetrated against HCWs and has labeled the surge in violence over the last decade as a healthcare emergency. Further, the WMA has called on the WHO and member states to take urgent action against WPV (2). The WHO has similarly acknowledged the impact of WPV on the physical and psychological health of HCWs and their subsequent job motivation, potentially compromising patient care and putting healthcare provision at risk (3).

Liu et al. recently quantified rates of WPV in healthcare and concluded that 62% of HCWs have been exposed to WPV. Liu et al. further noted that violence varied by geographic location and hospital department, with an increased prevalence in Asia and North America, and in the Emergency Department (ED) and psychiatric wards (4). Moreover, in their review articles exploring WPV specifically within an ED setting, Aljohani et al. postulated that 77% of ED staff members had been exposed to WPV, and Nikathil et al. suggested a rate of 36 violent patients for every 10,000 attendances (5, 6).

Aljohani et al. further explored the breakdown of WPV by role in their review and meta-analysis. They found that the majority of ED workers involved in WPV were nurses (55.7%) followed by physicians (36.5%), and that the primary instigators of WPV were family members (52%) followed by patients themselves (27%).

Many factors can lead to exposure to WPV, the causes of which have been extensively studied. The majority of studies note that nurses are the group most likely to be victims of abuse. Studies have also shown that increasing age, and a longer duration of employment are risk factors (7, 8). The association with gender and WPV is less clear, with some studies reporting men to experience higher rates of WPV, whereas a systematic review of studies in the African healthcare setting showed females to be at higher risk of WPV (8, 9).

Recent literature suggests that during the COVID-19 pandemic, there has been an alarming increase in WPV. For example, Chiroco et al. reported that WPV during this period was excessively high (10). Similarly, McGuire et al. found that, within one North American hospital referral region, violent incidents during the pandemic increased to 2.53/1000 visits compared to 1.13/1000 in the 3 months prior and 1.24/1000 in the year prior (11).

From a British perspective, the 2021 UK NHS Staff Survey revealed that within the previous 12 months, 14% of all NHS staff had experienced some sort of physical violence. A further 28% of the approximately 600,000 responses had experienced non-physical violence (12). Of frontline staff in hospitals with frequent patient contact, 19% were exposed to physical violence and 35% to non-physical violence. Results of the survey also indicated that only 42% of respondents reported an episode of violence. According to the survey, the groups most exposed to physical violence within the last 12 months were nursing and healthcare assistants (35.5%), followed by registered nurses and midwives (23.7%), and lastly medical and dental staff (11.4%). This was in contrast to harassment/bullying/verbal abuse where the groups most exposed were nurses and midwives (39.3%), followed by nursing axillary (38.0%), and finally medical and dental (34.2%). Two recent surveys conducted during the pandemic found that, over a one-month period, 30% of hospital doctors and 50% of General Practitioners had experienced verbal abuse, while the second survey found 35% of doctors had been subjected to verbal or physical abuse during the pandemic (13, 14).

The negative association of WPV with HCWs’ well-being has been extensively documented, particularly its role in depression, lifestyle changes, anxiety, PTSD and burnout (15–18). The 2022 GMC survey revealed that Emergency Medicine (EM) staff were at the highest risk of burnout, with 32% and 26% rates for trainees and trainers, respectively. Higher turnover of physicians has been reported in staff who have experienced WPV. This phenomenon is especially impactful in EM, which has a higher level of attrition than other specialities (19). Indeed, a review by McDermid et al. highlighted workplace aggression and violence as major themes in EM nurses (20). Moreover, a recent survey by the Royal College of Emergency Medicine reported that a large percentage of EM staff were considering altering working hours or leaving the field (21).

With this in mind, the aim of this study was to investigate and quantify trends in WPV within NHS EDs in the UK during the five-year period that included the COVID-19 pandemic. Since 2017 with the disbandment of NHS Protect, there has been no formalized national data collection on WPV for hospital trusts; therefore, this study serves to fill that knowledge gap, and provide justification for NHS trust-level policies to prevent WPV and support staff who fall victim to it.

## Methods

We sent Freedom of Information requests to all 152 trusts within England, Scotland, Wales and Northern Ireland that included a Type 1 department defined by NHS England as “A major Accident and Emergency (A&E) department – A consultant led 24-h service with full resuscitation facilities and designated accommodation for the reception of accident and emergency patients” and/or a Type 2 department defined as “a consultant led single speciality A&E service (e.g., ophthalmology, dental) with designated accommodation for the reception of emergency patients” (22).

We requested specific information on WPV, including physical assaults resulting in injury and non-injury, verbal abuse, sexual harassment/abuse, and other abuse not falling into these categories. Trusts were given a minimum of 80 working days to respond to the request. Trusts were requested to provide data on patient, relative or visitor violence on HCWs. Where patient/visitor on patient/visitor, HCW on HCW or HCW on patient/visitor information was provided, these were excluded from the analysis.

We requested aggregate data if we could not obtain exact numbers based on confidentiality exemptions. Where, due to low numbers and confidentiality concerns, only ranges were provided, the most conservative figure was used. For example, a range of five to ten was recorded as five.

We gathered monthly ED attendance data from publicly and freely available governmental sources; NHS England, Public Health Scotland, StatsWales and Northern Ireland Department of Health websites (23–26). For trusts that merged during the data collection period, data from the constituent trusts were consolidated for the new trusts. A&E attendances were defined by all 4 sources as ‘unplanned new’ or ‘unplanned review’ attendances whether they were admitted or not (22, 24–26).

Ratios were calculated by incidents reported compared to total attendance figures, and we only used attendance figures for the trusts reporting for that time period.

Descriptive data were collected and analyzed using Microsoft Excel®.

### Ethical statement

The UK Freedom of Information Act (2000) provides a right of access to information held by UK public authorities including the UK National Health Service. Individual trusts that responded to Freedom of Information (FOI) request replied with anonymized data in line with the UK Data Protection Act (2018) and UK GDPR legislation preventing release of third party data to the general public. Responsibility for the release of WPV data in the form of Freedom of Information requests to the general public is the responsibility of the individual trust releasing the data and identifiable information is exempt from FOI requests under Section 40 of the FOI act (27).

As analysis was run on publicly available data sets, in keeping with the principles of data protection and anonymity outlined above, no ethics committee approval was required.

### Statistical analysis

We conducted time series analysis using Stata/IC version 15. Yearly trend analysis was conducted using the Mann-Kenall test. We conducted a region-stratified analysis to detect variation by locality, and conducted subgroup analysis on specific categories of WPV (physical violence, sexual harassment/abuse).

Only complete years 2017–2021 were included in the analysis to prevent the possibility of seasonality impacts on the data.

To further elicit any effect of the pandemic, a monthly interrupted time series analysis (ITSA) using Prais-Winsten regression analysis was utilized as previously described (28). Our interrupted time series model was built around two key time points, the onset and offset of the UK lockdown. On March 16 2020, the UK government advised against all non-essential travel with a stay-at-home order and on May 10 2020, the first relaxation of lockdown rules occurred with an allowance to return to work. For all models, the distribution of residuals was inspected for approximation to normality. Due to the heteroscedastic nature of our data, we applied the generalized least squares model to our data over the ordinary least squares model, which can be statistically inefficient (29).

## Results

A total of 133 (88%) trusts responded to our FOI requests. Six trusts could not provide data due to confidentiality or storage issues (4%). Therefore, data were available from 127 (84%).

### Monthly analysis

At the time of manuscript submission, 75 trusts were able to provide monthly data. NHS records show that across the UK from January 2017 to March 2022, A&E attendances totaled 119,197,489. We received data for 58,459,667 or 49.0% of total A&E attendances throughout the UK during the period of interest (20–23). Monthly total WPV data are summarized, alongside total attendances for all UK trusts, in Table 1. Where possible, incident rates per 100,000 attendances have been calculated. It was not possible to calculate incident rates for trusts unable to provide monthly data.

**Table 1:** Monthly breakdown for attendances, total number of incidents and incidents/100,000 attendances.

Year		January	February	March	April	May	June	July	August	September	October	November	December
2017	Total Attendances	1,805,715	1,662,769	1,950,147	1,860,775	1,982,267	1,935,165	1,981,154	1,868,796	1,897,152	1,982,441	1,923,493	1,977,028
	Attendances used in analysis	817,322	751,191	878,699	832,462	887,156	865,008	890,248	847,358	862,749	895,174	865,524	882,721
	Total number of incidents	386	373	400	389	506	451	522	471	466	532	487	456
	Incidents Per 100,000	47.23	49.65	45.52	46.73	57.04	52.14	58.64	55.58	54.01	59.43	56.27	51.66
2018	Attendances	1,902,147	1,754,892	1,989,486	1,909,595	2,074,067	2,036,397	2,082,672	1,927,087	1,963,850	2,002,203	1,967,293	1,996,931
	Attendances used in analysis	860,534	796,720	904,703	905,676	986,889	964,708	993,237	915,225	932,055	967,879	947,685	960,460
	Total number of incidents	489	471	477	547	584	538	672	546	518	611	557	525
	Incidents Per 100,000	56.83	59.12	52.72	60.40	59.18	55.77	67.66	59.66	55.58	63.13	58.77	54.66
2019	Attendances	2,028,120	1,886,295	2,083,109	2,045,154	2,104,444	2,071,460	2,178,440	2,052,695	2,093,028	2,103,758	2,083,867	2,137,764
	Attendances used in analysis	975,337	908,542	1,006,168	986,528	1,019,658	1,001,016	1,079,212	1,013,857	1,030,689	1,068,055	1,058,143	1,084,223
	Total number of incidents	595	534	669	600	657	693	704	621	567	761	623	609
	Incidents Per 100,000	61.00	58.78	66.49	60.82	64.43	69.23	65.23	61.25	55.01	71.25	58.88	56.17
2020	Attendances	2,043,028	1,936,264	1,508,332	952,353	1,331,647	1,473,243	1,640,770	1,777,317	1,723,727	1,643,673	1,511,525	1,468,667
	Attendances used in analysis	1,034,513	979,505	772,818	489,804	683,653	758,508	847,582	914,614	889,392	846,044	781,524	754,101
	Total number of incidents	646	629	543	341	550	647	875	781	714	797	759	645
	Incidents Per 100,000	62.44	64.22	70.26	69.62	80.45	85.30	103.23	85.39	80.28	94.20	97.12	85.53
2021	Attendances	1,341,911	1,297,776	1,695,614	1,911,365	2,089,832	2,171,351	2,189,735	2,046,659	2,128,862	2,179,903	2,030,552	1,899,223
	Attendances used in analysis	696,800	677,111	884,473	998,423	1,090,989	1,131,944	1,138,672	1,067,079	1,112,143	1,134,834	1,059,200	991,056
	Total number of incidents	583	676	779	812	865	925	893	780	726	832	744	777
	Incidents Per 100,000	83.67	99.84	88.08	81.33	79.29	81.72	78.42	73.10	65.28	73.31	70.24	78.40
2022	Attendances	1,874,865	1,834,088	2,193,551	–	–	–	–	–	–	–	–	–
	Attendances used in analysis	980,734	958,185	1,143,155	–	–	–	–	–	–	–	–	–
	Total number of incidents	799	778	788	–	–	–	–	–	–	–	–	–
	Incidents Per 100,000	81.47	81.20	68.93	–	–	–	–	–	–	–	–	–

**Figure 1:**
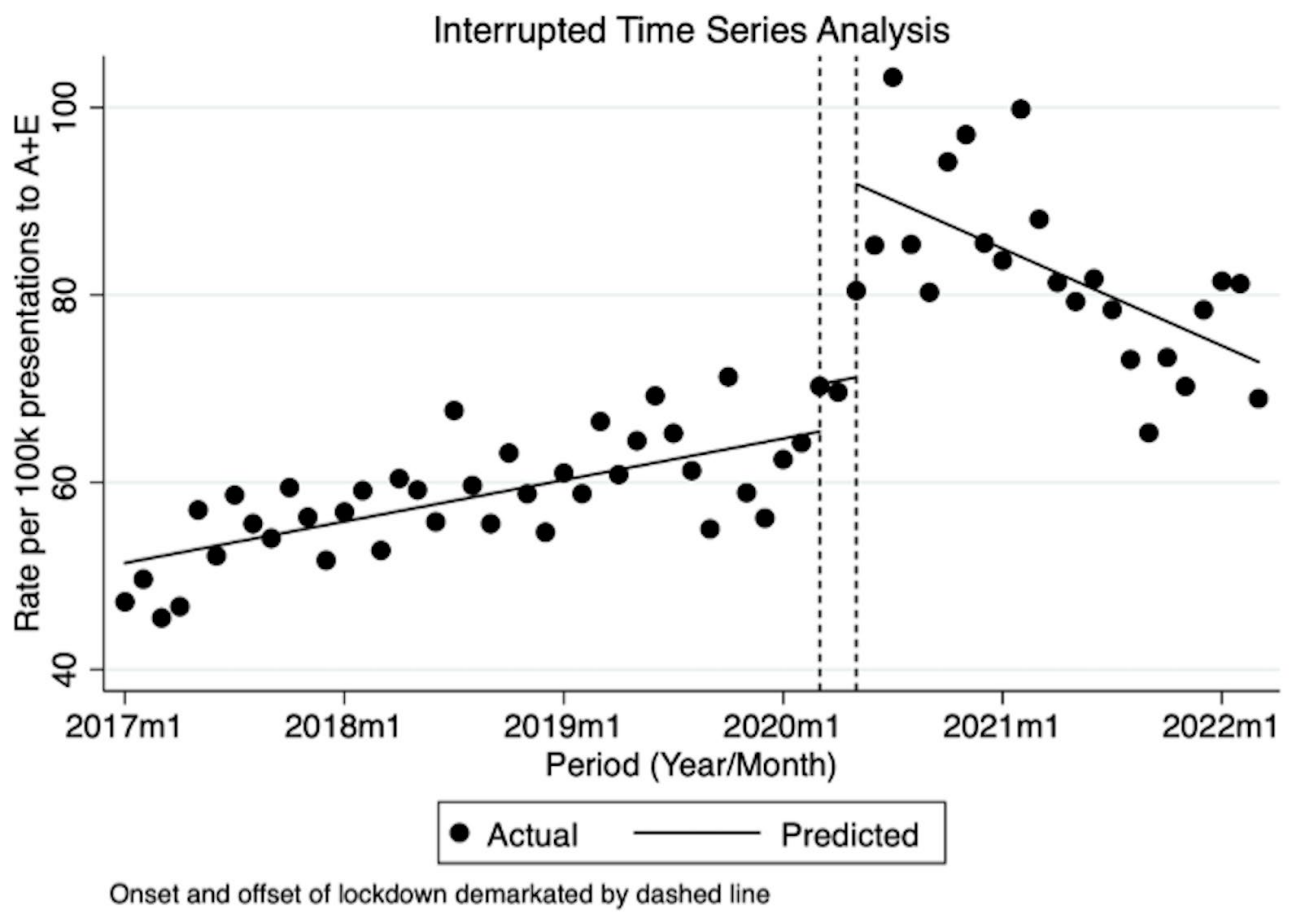
ITSA for monthly data of total WPV per 100,000 attendances.

Prior to March 2020, from January 2017, there was a statistically significant though small trend of an estimated increase of 0.37/100,000 attendances per month (95% CI 0.21/100,000–0.53/100,000 *p* < 0.0001) from an estimated 51.38/100,000 baseline determined by the model (95% CI 48.31/100,000–54.45/100,000). Incidents in March 2020, which coincided with the government-mandated stay at home lockdown order, increased by 5.06/100,000 (95% CI 1.59/100,000–8.53/100,000 *p* < 0.01) and remained high during the lockdown period with no detectable trend. During May 2020, when the government announced the lifting of lockdown and the return to work order, incidents increased by a further 20.63/100,000 (95% CI 9.39/100,000–31.87/100,000 *p* < 0.01). Following May 2020, although there was a decrease, this was not statistically significant-1.23/100,000 per month (95% CI-4.39/100,000–1.92/100,000 *p* = 0.48). These figures equate to a rise of approximately 50% in WPV over a roughly 2-year period. The levels of WPV at the end of our study, as predicted by the model, remain higher than pre-lockdown levels and far higher than the levels witnessed at the start of our study in January 2017. The graphical representation of the Prais-Winsten analysis is depicted in Figure 1.

### Yearly analysis

One hundred twenty seven out of one hundred fifty seven trusts provided yearly data. Of those, 99 provided complete data with exact figures (65%), 20 provided ranges only due to low numbers (13%), 7 were only able to provide certain time periods (5%), and 1 provided only a certain time period and range (0.7%).

From January 2017 to December 2021, out of a total of 113,294,985 ED visits, we received data covering 96,595,484 attendances allowing us to analyze 81.0% of all attendances within the UK during that time period.

The total number of incidents, rates per 100,000 attendances and *p* values for the presence of a statistically significant trend using Mann-Kendall analysis are detailed in Table 2.

**Table 2:** Total National and Regional WPV incidents, total number of incidents and incidents/100,000 attendances 2017–2021.

Region			2017	2018	2019	2020	2021	*P* value
United Kingdom		Attendances	22,826,902	23,606,620	24,868,134	19,010,546	22,982,783	–
		Attendances used in analysis	17,268,749	18,586,008	20,376,978	16,054,358	19,393,471	–
		Total number of incidents	9,760	10,600	12,369	12,749	15,253	–
		Incidents Per 100,000	56.52	57.03	60.70	79.41	78.65	0.086
England		Attendances	19,694,111	20,412,939	21,529,458	16,465,528	20,113,961	–
		Attendances used in analysis	14,497,515	15,765,155	17,436,396	13,801,926	16,864,078	–
		Total number of incidents	8,111	9,353	11,059	11,297	13,490	–
		Incidents Per 100,000	55.95	59.33	63.42	81.85	79.99	0.086
	East of England	Attendances	1,968,787	2,070,030	2,187,334	1,773,918	2,180,383	–
		Attendances used in analysis	1,368,076	1,494,371	1,681,380	1,390,604	1,713,705	–
		Total number of incidents	622	631	835	738	730	–
		Incidents Per 100,000	45.47	42.23	49.66	53.07	42.60	1.00
	London	Attendances	4,120,305	4,298,500	4,529,699	3,205,218	3,956,859	–
		Attendances used in analysis	2,590,128	2,683,210	2,969,267	2,227,230	2,737,494	–
		Total number of incidents	1,662	2,389	2,736	2,332	3,128	–
		Incidents Per 100,000	64.17	89.04	92.14	104.70	114.27	0.028
	Midlands	Attendances	3,510,359	3,636,878	3,874,364	2,956,190	3,653,149	–
		Attendances used in analysis	2,462,054	2,599,894	2,777,899	2,096,936	2,637,310	–
		Total number of incidents	1,917	1,724	1,746	1,608	1,697	–
		Incidents Per 100,000	77.86	66.31	62.85	76.68	64.35	0.46
	North East and Yorkshire	Attendances	3,365,758	3,470,177	3,681,222	2,808,001	3,477,898	–
		Attendances used in analysis	2,500,029	2,788,484	2,977,117	2,268,802	2,824,199	–
		Total number of incidents	846	1,021	1,093	1,388	1,745	–
		Incidents Per 100,000	33.84	36.61	36.71	61.18	61.79	0.068
	North West	Attendances	3,159,419	3,205,324	3,314,320	2,547,068	3,080,742	–
		Attendances used in analysis	2,087,767	2,537,634	2,877,877	2,409,375	2,912,975	–
		Total number of incidents	856	1,159	1,646	1,856	2,526	–
		Incidents Per 100,000	41.00	45.67	57.19	77.03	86.72	0.028
	South East	Attendances	2,477,965	2,528,797	2,692,563	2,187,533	2,622,513	–
		Attendances used in analysis	1,818,602	1,856,272	2,268,061	1,927,039	2,318,907	–
		Total number of incidents	1,127	1,225	1,726	1,947	2,302	–
		Incidents Per 100,000	61.97	65.99	76.10	101.04	99.27	0.086
	South West	Attendances	1,670,859	1,805,290	1,884,795	1,481,940	1,719,488	–
		Attendances used in analysis	1,670,859	1,805,290	1,884,795	1,481,940	1,719,488	–
		Total number of incidents	1,080	1,204	1,277	1,428	1,362	–
		Incidents Per 100,000	64.64	66.69	67.75	96.36	79.21	0.086
Scotland		Attendances	1,348,163	1,367,894	1,436,432	1,085,365	1,239,818	–
		Attendances used in analysis	1,341,541	1,361,085	1,429,422	1,080,833	1,235,709	–
		Total number of incidents	643	503	490	560	490	–
		Incidents Per 100,000	47.93	36.96	34.28	51.81	39.65	1.00
Wales		Attendances	807,543	814,684	843,668	666,417	734,434	–
		Attendances used in analysis	807,543	814,684	843,668	666,417	734,434	–
		Total number of incidents	812	458	546	628	897	–
		Incidents Per 100,000	100.55	56.22	64.72	94.24	122.13	0.46
Northern Ireland		Attendances	785,504	811,313	841,588	632,454	692,828	–
		Attendances used in analysis	785,504	645,084	667,492	505,182	559,250	–
		Total number of incidents	194	286	274	264	376	–
		Incidents Per 100,000	31.18	44.34	41.05	52.26	67.23	0.086

Throughout the UK, the rate of WPV per 100,000 attendances rose from 57/100,000 in 2017 to 79/100,000 in 2021, an increase of 39% over 5 years. Initial data collection from the first 3 months of 2022 suggests a rate of 77/100,000. The largest yearly change was seen during 2019–2020 from 61/100,000 to 79/100,000.

Wales had the highest number of incidents per 100,000 attendances, reaching a peak of 122/100,000 in 2021; initial data from 2022 suggests a reduction to 77/100,000.

On a country level, no statistically significant detectable trend existed for any of the Home Nations. However, the UK as a whole, as well as England and Northern Ireland are approaching significance with the presence of a trend (*p* < 0.1).

**Figure 2:**
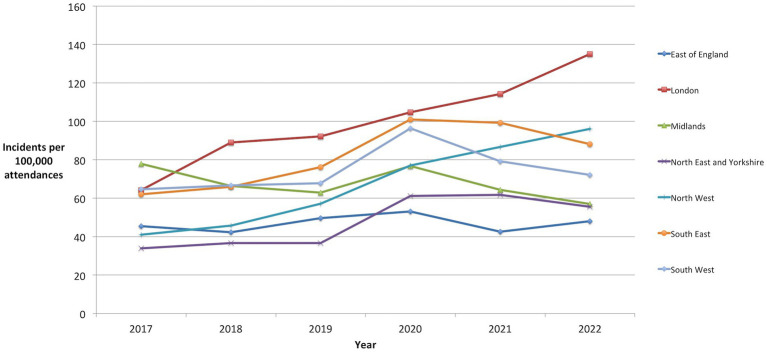
WPV incidents per total 100,000 attendances within regions of England.

Regional analysis with geographical groupings as described by NHS England revealed a considerable increase in incidents per 100,000 in 5 regions – South East (60%), London (78%), North East and Yorkshire (83%), North West (112%) and South West (23%) and a decrease in 2 regions – Midlands (-17%) and East of England (-6%) as detailed in Table 2 and depicted graphically in Figure 2.

Trend analysis showed statistically significant rising trends in London and the North-West of England (*p* < 0.05). Regions approaching statistically significant trends were the North-East England, South-East England and the South-West England (p < 0.1). Mann-Kendall analyses for the presence of a statistically significant trend over the 5 years are also presented in Table 2.

## Subgroup analysis

### Physical abuse

Eighty one trusts provided data on physical violence. We received data covering 80,872,878 ED attendances, 71.3% of all ED attendances during that period.

A breakdown of physical violence is shown in Table 3. Overall from 2017 to 2021, there was an increase of 27% in incidents per 100,000 (18/100,000 to 23/100,000) down 9% from a peak of 25/100,000 in 2020. There were increases in physical abuse rates per 100,000 in England (26%), Wales (61%) and Northern Ireland (159%) and a decrease in Scotland (19%).

**Table 3:** National and Regional physical violence incidents, total number of incidents and per 100,000 attendances.

Region			2017	2018	2019	2020	2021	*P* Value
United Kingdom		Total number of incidents	2,750	2,905	3,409	3,616	4,000	–
		Incidents Per 100,000	18.22	17.79	18.99	25.46	23.13	0.31
England		Total number of incidents	2,333	2,585	3,045	3,204	3,488	–
		Incidents Per 100,000	18.29	18.45	19.62	25.97	22.93	0.13
	East of England	Total number of incidents	187	192	263	191	225	–
		Incidents Per 100,000	13.67	12.85	15.64	13.74	13.13	1.00
	London	Total number of incidents	430	545	616	545	640	–
		Incidents Per 100,000	17.97	21.98	22.30	26.15	24.79	0.086
	Midlands	Total number of incidents	482	396	464	457	451	–
		Incidents Per 100,000	23.01	18.59	20.35	26.33	20.65	1.00
	North East and Yorkshire	Total number of incidents	225	254	304	299	437	–
		Incidents Per 100,000	9.00	9.11	10.21	13.18	15.47	0.043
	North West	Total number of incidents	180	274	352	530	796	–
		Incidents Per 100,000	13.01	14.96	17.00	30.15	35.65	0.028
	South East	Total number of incidents	457	490	678	745	627	–
		Incidents Per 100,000	25.13	26.40	29.89	38.66	27.04	0.22
	South West	Total number of incidents	357	375	368	437	312	–
		Incidents Per 100,000	25.80	24.33	22.74	34.15	20.99	0.46
Scotland		Total number of incidents	180	114	136	148	145	–
		Incidents Per 100,000	15.01	9.36	10.64	15.36	13.08	1.00
Wales		Total number of incidents	211	146	169	215	306	–
		Incidents Per 100,000	33.46	22.96	25.66	41.03	53.90	0.22
Northern Ireland		Total number of incidents	26	60	59	49	61	–
		Incidents Per 100,000	5.76	12.79	12.04	12.99	14.89	0.096

North-West England recorded a statistically significant trend for increased physical violence (*p* < 0.05), London, North-East England and Yorkshire and Northern Ireland all approached significance (p < 0.1).

Further sub-group analysis on physical violence and sexual abuse and harassment from January 2017–March 2022 can be found in the supplementary files.

Two trusts changed their reporting procedures during the request time period. These were Northern Lincolnshire and Goole NHS Foundation Trust (January 2018) and Manchester University NHS Foundation Trust (March 2018). Data derived from the old methods were excluded due to significant variance in incidents pre and post reporting method change.

## Discussion

This study presents the results of a large, multicentre investigation of WPV within EDs, at a regional and country level. To our knowledge, this is the first study to investigate the country-wide associations of WPV, the COVID-19 pandemic and the subsequent lockdown.

Of great concern is that our study has shown that WPV has persisted above pre-pandemic levels both in the total number of incidents and per attendance, a change that is statistically significant. While there were small detectable increases prior to COVID-19, there was a notable increase in violence at the start of the lockdown period and a further drastic and significant increase when the lockdown was eased.

Our results corroborate the results of an Israeli study noting HCWs’ perception of increasing WPV during the pandemic (30). Our data are also in keeping with the findings of the McGuire et al. study, which demonstrated an increase in WPV during COVID-19. Albeit, the increase in WPV described in our study throughout the COVID period was 50%, compared to 200% in the paper by McGuire et al. (8) A similar increase in WPV has been documented in primary care within the UK, with violent incidents doubling in 5 years (31).

Our study also demonstrates regional variation in WPV, both within the Home Nations as well as the English regions. For example, the rate of WPV in Scotland decreased over the COVID period, as did WPV in the Midlands and East of England regions. By contrast, WPV increased significantly in London (64.17/100,000 to 114.27 *p* < 0.05) and the North West of England (41.00/100,000 to 86.72/100,000 *p* < 0.05). Furthermore, physical violence increased significantly in the North West of England (13.01/100,000 to 35.65/100,000 p < 0.05). Although the factors contributing to this regional variation are beyond the scope of this study, it represents an interesting area for further research. Nonetheless, policymakers should be mindful of areas with an increased incidence of violence and prioritize these areas for the implementation of anti-violence policies and campaigns.

Additionally, we have identified certain regions where there is an increasing trend of physical violence. Specific policies designed to mitigate the effect of physical abuse, where strategies can differ from other types of abuse, should be targeted to these regions at risk (32).

As previously noted in the NHS staff survey, not all WPV incidents are reported. Indeed, 25–50% of all incidents go unreported (12). Therefore, the true incidence and, by extension, the impact of WPV is likely to be higher than that posited in this paper.

Academics have hypothesized several potential precipitants of WPV against HCWs. Previous studies identified the following as potential triggers: long wait times to see a clinician or receive medication, a shortage of staff, exposure to pain, high-stress levels and feelings of anger, frustration and unmet patient needs (33, 34). These proposed triggers provide an interesting comparison to our own study, which spanned the COVID-19 pandemic. NHS data revealed the height of the pandemic to be a period of low attendance and improved wait times within EDs (35). On that basis, therefore, one would expect violence levels to have fallen as the aforementioned precipitants were largely addressed, inadvertently or otherwise. This, however, was not the finding in our study. Therefore, explaining the increase in violence is not as simple as blaming the pandemic. By logical extension, a ‘return-to-normal’ cannot be expected to be the sole antidote.

More recently the NHS has been experiencing a retention crisis with healthcare workers of all disciplines leaving the field, including 34,000 nurses leaving their posts and 10,000 doctors giving up their license to practice in 2022 (36, 37). Worryingly in a survey of 4,500 junior doctors in the UK a third planned to work abroad in the next 12 months, and 40% would leave the NHS as soon as they found another job (38). Job dissatisfaction has recently manifested itself with the most widespread industrial action within the NHS. A recent meta-analysis of 6,400 nurses showed that WPV was associated with nursing turnover (39). Furthermore, recent studies concluded that perceived organizational support reduced the effect of WPV upon turnover (40, 41).

For a nationalized health service like the NHS, which already suffers from 133,000 vacancies (9.7% of the total workforce) in England alone, the impact of increasing violence against HCWs could be catastrophic toward this retention crisis (42). The negative psychological effects, such as stress and burnout, of WPV on HCWs have been well documented (15–18). With a steep rise in WPV, it is highly likely that the collective morale of the workforce has plummeted, and their well-being suffered. Within a cohort of ED nurses, there was shown to be a direct correlation between experiencing WPV and reduced job satisfaction and increased turnover (43).

Additionally, poor staff morale and widespread rota gaps could lead to worsening service provision capability, which can have significant detrimental effects on patient care and outcomes, as well as triggering further WPV; a vicious cycle ensues.

Previously, efforts to reduce the incidence of WPV and its effects on HCWs have been targeted toward security interventions, support groups and staff training, which have all been discussed as positive and useful interventions (44–46). In view of the findings of our study, these and other interventions to safeguard staff would benefit from increased resource allocation and implementation within the NHS.

In 2020 NHS England released the Violence Prevention and Reduction standard (47). Despite this, WPV remains concerningly high, and our results should provide policymakers with evidence that further efforts are needed to halt and reverse this worrying trend. In the current climate of widespread industrial action over working conditions within the NHS, WPV should be an area that is focused on nationally in light of this study’s findings. We further suggest that reducing WPV and increasing support for WPV victims would have an immediate effect on staff morale and well-being, and improve efforts at reducing staff turnover. This should be done expeditiously in view of the current NHS staffing crisis.

## Limitations

Due to a large number of trusts and incident reporting systems, there is the potential for a wide variation in the way incidents have been reported and recorded by each individual trust, which could impact results. Twenty trusts were unable to provide exact figures; however, as this was the case only if incident numbers were low, the figures are judged to have a small impact on the overall validity of the results and would lead to an under-represented number of WPV incidents. Further due to the anonymized nature of the data sets released *via* the FOI requests and publicly available attendance data, we did not receive any demographic data on any of the participants involved in the violence. Therefore, specific commentary on the representativeness of the data to the entire UK population is limited. Due to the small number of time points, particularly in regard to yearly data analysis, the potential for Type II statistical errors with no trend detected is elevated. Further data gathering covering more time points in the future could mitigate this potential.

## Conclusion

Our study has demonstrated that there has been a dramatic rise of 50% in the number of WPV incidents recorded throughout 2017–2022 in the UK, with significant, drastic increases over the COVID lockdown period. WPV critically impacts upon staff morale and well-being, resulting in problems related to staff retention, recruitment and the efficient running of the department. Ultimately, patient outcomes are put at risk. There is some regional and geographical variance in rates of violence, the cause of which is uncertain. Further investigation is required.

We believe that the findings of this study highlight an emergency in WPV, and should serve as a precipitant for targeted reduction strategies. This paper provides evidence to encourage healthcare leaders to accelerate the formulation and implementation of policies to safeguard HCWs in a period of particularly low morale and well-being as shown by large-scale industrial action within the workforce. With the ongoing retention crisis within the NHS, a reduction in WPV will provide an immediate boost to an increasingly dejected and demoralized workforce.

## Data availability statement

The original contributions presented in the study are included in the article/Supplementary material, further inquiries can be directed to the corresponding author.

## Author contributions

ND contributed to conceptualization, methodology, and data collection. TL contributed to software and formal statistical analysis. All authors contributed to manuscript draft and review. All authors have read and agreed to the published version of the manuscript.

## Conflict of interest

The authors declare that the research was conducted in the absence of any commercial or financial relationships that could be construed as a potential conflict of interest.

## Publisher’s note

All claims expressed in this article are solely those of the authors and do not necessarily represent those of their affiliated organizations, or those of the publisher, the editors and the reviewers. Any product that may be evaluated in this article, or claim that may be made by its manufacturer, is not guaranteed or endorsed by the publisher.
