# Supplementary material for: Incidence and trends in workplace violence within emergency departments in the United Kingdom 2017–2022: an observational time series analysis
Source: Front Public Health. 2023 Jun 28;11:1211471. doi: 10.3389/fpubh.2023.1211471 (PMC10336324; doi:10.3389/fpubh.2023.1211471)
Supplement: Supplementary file 1 [file Data_Sheet_1.PDF]

Supplementary Material

Incidence and trends in Workplace Violence within Emergency Departments in the United Kingdom 2017-2022: An observational time series analysis

Neil Donald[1*], Tim Lindsay[2,3]

[1]Dartford and Gravesham NHS Trust, Darenth Wood Road, Dartford, DA2 8DA
[2]Department of Trauma and Orthopaedics, London North West University Hospitals NHS Trust, UK
[3] Imperial College London, UK

Correspondence:
Neil Donald
n.donald@doctors.org.uk

Supplementary Figure 1. WPV Incidents per 100,000 attendances within the Home Nations of the United Kingdom.

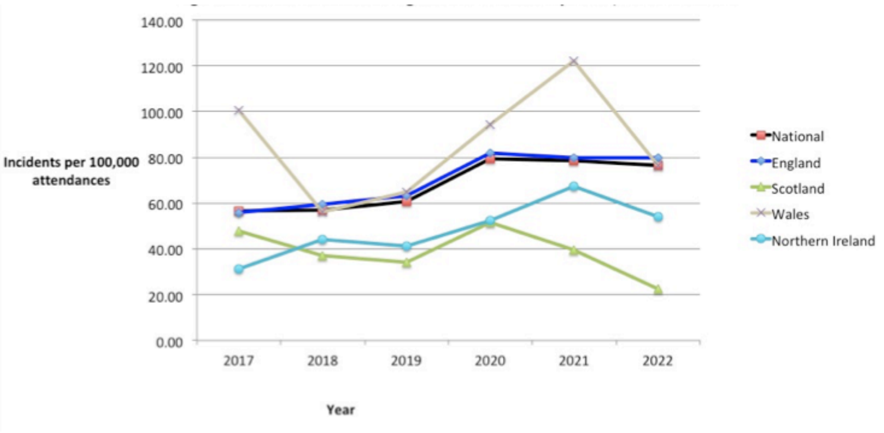

Supplementary Figure 2. Physical WPV incidents per 100,000 attendances within the Home Nations of the United Kingdom

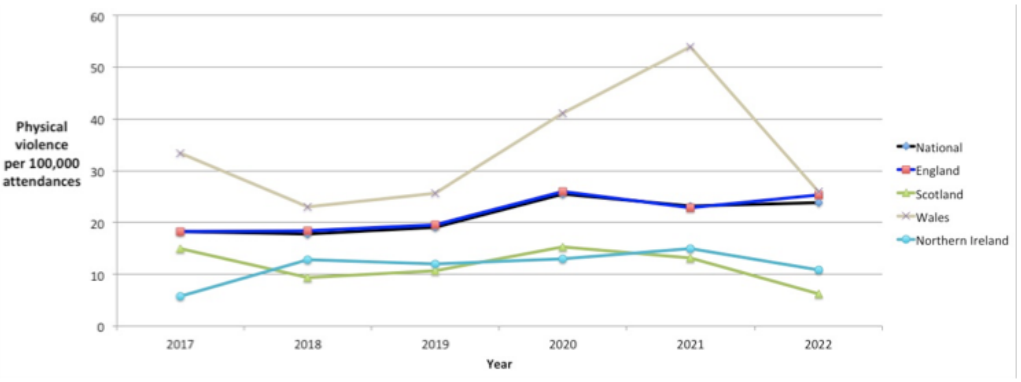

Supplementary Figure 3. Physical WPV incidents per 100,000 attendances within regions of England

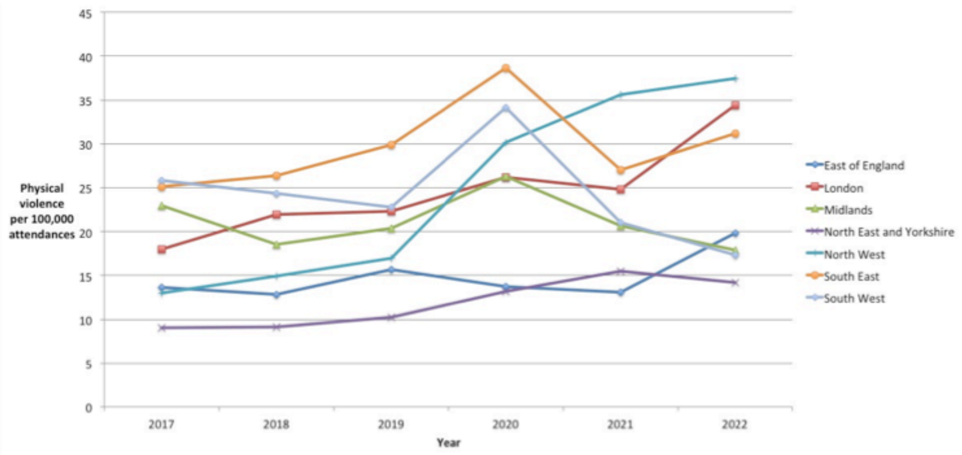

Supplementary Table 1. National Injuries and Non-injuries incidents, gross and per 100,000 attendances

Region			2017	2018	2019	2020	2021	P Value
**United Kingdom**	Injuries	Gross	890	1,018	1,071	1,113	1,108	
		Incidents Per 100,000	7.72	8.33	8.23	11.09	9.09	0.1296
	Non-injuries	Gross	1,220	1,199	1,398	1,378	1,417	
		Incidents Per 100,000	10.58	9.81	10.75	13.73	11.62	0.3122
	Proportion of incidents causing injuries		42%	46%	43%	45%	44%	

Supplementary Table 2. National sexual abuse incidents, total number of incidents and per 100,000 incidents

Region		2017	2018	2019	2020	2021	P value
**United Kingdom**	Total number of incidents	74	108	99	137	156	
	Incidents Per 100,000	0.60	0.79	0.67	1.16	1.08	0.15
